# *In Vitro* Activity of Cefepime-Taniborbactam and Comparators against Clinical Isolates of Gram-Negative Bacilli from 2018 to 2020: Results from the Global Evaluation of Antimicrobial Resistance via Surveillance (GEARS) Program

**DOI:** 10.1128/aac.01281-22

**Published:** 2022-12-21

**Authors:** James A. Karlowsky, Meredith A. Hackel, Mark G. Wise, David A. Six, Tsuyoshi Uehara, Denis M. Daigle, Susan M. Cusick, Daniel C. Pevear, Greg Moeck, Daniel F. Sahm

**Affiliations:** a IHMA, Schaumburg, Illinois, USA; b Department of Medical Microbiology and Infectious Diseases, Max Rady College of Medicine, University of Manitoba, Winnipeg, Manitoba, Canada; c Venatorx Pharmaceuticals, Inc., Malvern, Pennsylvania, USA

**Keywords:** β-lactamase inhibitor, *Enterobacterales*, Gram-negative, *Pseudomonas aeruginosa*, taniborbactam, carbapenem resistant, multidrug resistant

## Abstract

Taniborbactam is a novel cyclic boronate β-lactamase inhibitor in clinical development in combination with cefepime. We assessed the *in vitro* activity of cefepime-taniborbactam and comparators against a 2018–2020 collection of *Enterobacterales* (*n *= 13,731) and Pseudomonas aeruginosa (*n *= 4,619) isolates cultured from infected patients attending hospitals in 56 countries. MICs were determined by CLSI broth microdilution. Taniborbactam was tested at a fixed concentration of 4 μg/mL. Isolates with cefepime-taniborbactam MICs of ≥16 μg/mL underwent whole-genome sequencing. β-lactamase genes were identified in meropenem-resistant isolates by PCR/Sanger sequencing. Against *Enterobacterales*, taniborbactam reduced the cefepime MIC_90_ value by >64-fold (from >16 to 0.25 μg/mL). At ≤16 μg/mL, cefepime-taniborbactam inhibited 99.7% of all *Enterobacterales* isolates; >97% of isolates with multidrug-resistant (MDR) and ceftolozane-tazobactam-resistant phenotypes; ≥90% of isolates with meropenem-resistant, difficult-to-treat-resistant (DTR), meropenem-vaborbactam-resistant, and ceftazidime-avibactam-resistant phenotypes; 100% of VIM-positive, AmpC-positive, and KPC-positive isolates; 98.7% of extended-spectrum β-lactamase (ESBL)-positive; 98.8% of OXA-48-like-positive; and 84.6% of NDM-positive isolates. Against P. aeruginosa, taniborbactam reduced the cefepime MIC_90_ value by 4-fold (from 32 to 8 μg/mL). At ≤16 μg/mL, cefepime-taniborbactam inhibited 97.4% of all P. aeruginosa isolates; ≥85% of isolates with meropenem-resistant, MDR, and meropenem-vaborbactam-resistant phenotypes; >75% of isolates with DTR, ceftazidime-avibactam-resistant, and ceftolozane-tazobactam-resistant phenotypes; and 87.4% of VIM-positive isolates. Multiple potential mechanisms, including carriage of IMP, certain alterations in PBP3, permeability (porin) defects, and possibly, upregulation of efflux were present in most isolates with cefepime-taniborbactam MICs of ≥16 μg/mL. We conclude that cefepime-taniborbactam exhibited potent *in vitro* activity against *Enterobacterales* and P. aeruginosa and inhibited most carbapenem-resistant isolates, including those carrying serine carbapenemases or NDM/VIM metallo-β-lactamases (MBLs).

## INTRODUCTION

The prevalence of carbapenem-resistant *Enterobacterales* (CRE) and carbapenem-resistant Pseudomonas aeruginosa (CRPA) continues to increase worldwide (1–4). The WHO classifies CRE and CRPA as critical, priority 1 pathogens (5), and the CDC lists CRE and multidrug-resistant (MDR; frequently carbapenem-resistant) P. aeruginosa as urgent and serious threats (6). Development of β-lactam/β-lactamase inhibitor combinations with activity against MDR *Enterobacterales* and P. aeruginosa, including those producing metallo-β-lactamases (MBLs) is critical. MBL genes are mobile, and the enzymes are structurally diverse, frequently conferring a pan-β-lactam-resistant phenotype and contributing significantly to MDR phenotypes, leaving clinicians with few, often toxic and less efficacious, treatment options for their patients. There are currently no approved β-lactam/β-lactamase inhibitor combinations that cover MBL-producing Gram-negative bacilli. An investigational combination of cefepime and taniborbactam, an inhibitor of both serine and MBL enzymes, has completed phase 3 clinical development for the treatment of patients with complicated urinary tract infection (7, 8).

Taniborbactam is a boronic acid-containing inhibitor of Ambler class A, C, and D (serine) β-lactamases and class B MBLs, including VIM and NDM but not IMP (9, 10). Taniborbactam acts as a reversible, covalent inhibitor of serine β-lactamases and as a competitive inhibitor of MBLs (9, 10). Taniborbactam restores the activity of cefepime against many MDR and difficult-to-treat resistant (DTR) organisms, including cephalosporin-resistant *Enterobacterales* and P. aeruginosa, and CRE and CRPA carrying MBLs (9–16).

The current study assessed the *in vitro* activity of cefepime-taniborbactam and comparator agents against a global collection of clinical isolates of *Enterobacterales* and P. aeruginosa collected from 2018 to 2020 as part of the Global Evaluation of Antimicrobial Resistance via Surveillance (GEARS) program.

## RESULTS

### *Enterobacterales*: all isolates and isolates with antimicrobial-resistant phenotypes.

The 13,731 *Enterobacterales* isolates tested were 26.0% levofloxacin resistant, 23.4% ceftazidime resistant, 17.4% cefepime resistant, 15.4% gentamicin resistant, 10.9% piperacillin-tazobactam resistant, and 4.6% meropenem resistant (see Table S1 in the supplemental material). Overall, 31.6% (4,335/13,731) of isolates had an extended-spectrum β-lactamase (ESBL) phenotype (Table 1). MDR and DTR phenotypes were identified in 13.0% and 4.5% of *Enterobacterales* isolates, respectively.

**TABLE 1:** *In vitro* activity of cefepime-taniborbactam and comparator agents against isolates of Enterobacterales with antimicrobial-resistant phenotypes

Phenotype (no. of isolates; % of total)	Antimicrobial agent	MIC (μg/mL)			MIC interpretation		
		MIC_50_	MIC_90_	MIC range	Susceptible (%)	Intermediate (%)	Resistant (%)
All isolates (13,731; 100)	Cefepime-taniborbactam^*a*^	0.06	0.25	≤0.008 to >16	99.7	NA^*b*^	0.3
	Ceftazidime-avibactam	≤0.12	0.5	≤0.12 to >16	97.8	NA	2.2
	Ceftolozane-tazobactam	0.5	8	≤0.25 to >8	87.1	2.4	10.5
	Meropenem-vaborbactam	≤0.06	0.12	≤0.06 to >16	97.4	0.3	2.2
	Piperacillin-tazobactam	≤4	128	≤4 to >128	84.2	4.9	10.9
							
ESBL phenotype (4,335; 31.6)^*c*^	Cefepime-taniborbactam	0.12	1	≤0.008 to >16	99.1	NA	0.9
	Ceftazidime-avibactam	0.25	2	≤0.12 to >16	93.1	NA	6.9
	Ceftolozane-tazobactam	2	>8	≤0.25 to >8	59.6	7.1	33.3
	Meropenem-vaborbactam	≤0.06	2	≤0.06 to >16	91.9	1.0	7.0
	Piperacillin-tazobactam	16	>128	≤4 to >128	55.5	13.5	31.0
							
Cefepime resistant (2,390; 17.4)	Cefepime-taniborbactam	0.25	4	≤0.008 to >16	98.3	NA	1.7
	Ceftazidime-avibactam	0.5	>16	≤0.12 to >16	88.3	NA	11.7
	Ceftolozane-tazobactam	2	>8	≤0.25 to >8	52.1	5.2	42.7
	Meropenem-vaborbactam	≤0.06	16	≤0.06 to >16	85.6	1.8	12.6
	Piperacillin-tazobactam	32	>128	≤4 to >128	48.7	12.0	39.3
							
MDR phenotype (1,781; 13.0)^*d*^	Cefepime-taniborbactam	0.25	4	0.016 to >16	97.9	NA	2.1
	Ceftazidime-avibactam	0.5	>16	0.12 to >16	83.9	NA	16.1
	Ceftolozane-tazobactam	>8	>8	0.25 to >8	32.8	5.1	62.2
	Meropenem-vaborbactam	≤0.06	>16	0.06 to >16	80.3	2.6	17.1
	Piperacillin-tazobactam	128	>128	4 to >128	30.7	12.3	57.0
							
Piperacillin-tazobactam resistant (1,498; 10.9)	Cefepime-taniborbactam	0.5	4	0.016 to >16	97.5	NA	2.5
	Ceftazidime-avibactam	1	>16	≤0.12 to >16	81.7	NA	18.3
	Ceftolozane-tazobactam	>8	>8	≤0.25 to >8	20.5	5.1	74.9
	Meropenem-vaborbactam	≤0.06	>16	≤0.06 to >16	77.0	2.9	20.2
	Piperacillin-tazobactam	>128	>128	128 to >128	0	0	100
							
Ceftolozane-tazobactam resistant (1,444; 10.5)	Cefepime-taniborbactam	0.5	4	0.016 to >16	97.4	NA	2.6
	Ceftazidime-avibactam	1	>16	≤0.12 to >16	79.5	NA	20.5
	Ceftolozane-tazobactam	>8	>8	8 to >8	0	0	100
	Meropenem-vaborbactam	0.12	>16	≤0.06 to >16	75.9	3.1	21.0
	Piperacillin-tazobactam	>128	>128	≤4 to >128	6.3	16.0	77.7
							
Meropenem resistant (637; 4.6)	Cefepime-taniborbactam	1	8	0.016 to >16	94.5	NA	5.5
	Ceftazidime-avibactam	4	>16	≤0.12 to >16	59.0	NA	41.0
	Ceftolozane-tazobactam	>8	>8	1 to >8	1.1	1.6	97.3
	Meropenem-vaborbactam	8	>16	≤0.06 to >16	44.9	7.2	47.9
	Piperacillin-tazobactam	>128	>128	≤4 to >128	0.2	1.9	98.0
							
DTR phenotype (623; 4.5)^*e*^	Cefepime-taniborbactam	1	16	0.016 to >16	94.4	NA	5.6
	Ceftazidime-avibactam	4	>16	≤0.12 to >16	61.2	NA	38.8
	Ceftolozane-tazobactam	>8	>8	2 to >8	0	0	100
	Meropenem-vaborbactam	8	>16	≤0.06 to >16	47.8	6.3	45.9
	Piperacillin-tazobactam	>128	>128	32 to >128	0	1.4	98.6
							
Meropenem-vaborbactam resistant (305; 2.2)	Cefepime-taniborbactam	2	16	0.12 to >16	90.2	NA	9.8
	Ceftazidime-avibactam	>16	>16	≤0.12 to >16	32.8	NA	67.2
	Ceftolozane-tazobactam	>8	>8	2 to >8	0.3	0.3	99.3
	Meropenem-vaborbactam	>16	>16	16 to >16	0	0	100
	Piperacillin-tazobactam	>128	>128	32 to >128	0	1.0	99.0
							
Ceftazidime-avibactam resistant (299; 2.2)	Cefepime-taniborbactam	1	>16	0.03 to >16	89.6	NA	10.4
	Ceftazidime-avibactam	>16	>16	16 to >16	0	NA	100
	Ceftolozane-tazobactam	>8	>8	≤0.25 to >8	0.3	0.7	99.7
	Meropenem-vaborbactam	>16	>16	≤0.06 to >16	24.8	6.7	68.6
	Piperacillin-tazobactam	>128	>128	≤4 to >128	4.4	4.0	91.6

a For comparative purposes only, percent susceptible and percent resistant for cefepime-taniborbactam correspond to the percentage of isolates inhibited at ≤16 μg/mL and ≥32 μg/mL, respectively.

b NA, not applicable.

c ESBL phenotype screening criteria were modified from those published by CLSI (31) for the purpose of this study, with an ESBL-positive phenotype assigned to isolates of *Enterobacterales* with ceftazidime and/or cefepime MICs of ≥2 μg/mL.

d An MDR phenotype was assigned to isolates resistant to at least one agent from ≥3 of the following antimicrobial agent classes: aminoglycosides (gentamicin), β-lactam combination agents (piperacillin-tazobactam, ceftazidime-avibactam, ceftolozane-tazobactam, meropenem-vaborbactam), carbapenems (meropenem or imipenem), cephems (ceftazidime, cefepime), and fluoroquinolones (levofloxacin or ciprofloxacin).

e DTR isolates were identified using the definition of Kadri et al. (42) as isolates intermediate or resistant to fluoroquinolones (levofloxacin) and all β-lactams, including carbapenems and piperacillin-tazobactams but excluding ceftazidime-avibactam, ceftolozane-tazobactam, and meropenem-vaborbactam.

Against all *Enterobacterales* isolates, the cefepime-taniborbactam MIC_50_ and MIC_90_ were 0.06 and 0.25 μg/mL, respectively; 99.7% of isolates were inhibited at ≤16 μg/mL (Table 1). At cefepime-taniborbactam concentrations of ≤8 μg/mL, ≤4 μg/mL, and ≤2 μg/mL, 99.5%, 99.0%, and 98.1%, respectively, of all *Enterobacterales* isolates were inhibited (data not shown). The addition of taniborbactam reduced the cefepime MIC_90_ value by >64-fold, from >16 μg/mL to 0.25 μg/mL, for all *Enterobacterales* isolates (Table S1). Percent susceptible values were 97.8% for ceftazidime-avibactam, 97.4% for meropenem-vaborbactam, and 87.1% for ceftolozane-tazobactam against all *Enterobacterales* isolates (Table 1).

Cefepime-taniborbactam MIC_90_ values varied by 16-fold across the *Enterobacterales* species tested (Table S2). The MIC_90_ values were 0.06 μg/mL for Klebsiella oxytoca and Proteus vulgaris; 0.12 μg/mL for Citrobacter freundii complex, Escherichia coli, Klebsiella aerogenes, Morganella morganii, Proteus mirabilis, Providencia rettgeri, Providencia stuartii, and Serratia liquifaciens; 0.25 μg/mL for Serratia marcescens; 0.5 μg/mL for Enterobacter cloacae complex; and 1 μg/mL for Klebsiella pneumoniae. As anticipated, MIC_50_ values showed less variation (4-fold [0.016 to 0.06 μg/mL]) across all species of *Enterobacterales*.

Cefepime-taniborbactam at ≤16 μg/mL inhibited 99.1% of ESBL phenotype, 98.3% of cefepime-resistant, 97.9% of MDR, 97.5% of piperacillin-tazobactam-resistant, 97.4% of ceftolozane-tazobactam-resistant, 94.5% of meropenem-resistant, 94.4% of DTR, 90.2% of meropenem-vaborbactam-resistant, and 89.6% of ceftazidime-avibactam-resistant isolates of *Enterobacterales* (Table 1). Cefepime-taniborbactam inhibited a greater percentage of isolates with each of the nine resistance phenotypes studied than ceftazidime-avibactam, meropenem-vaborbactam, and ceftolozane-tazobactam.

### *Enterobacterales*: isolates with antimicrobial-resistant genotypes.

Among all *Enterobacterales* isolates that qualified for molecular characterization (*n *= 1,265), 50.0% were carbapenemase positive, including 235 MBL-positive isolates (18.6%). The MBL-positive isolates comprised 207 (88.1%) NDM-positive; 22 (9.4%) VIM-positive, 5 (2.1%) IMP-positive, and 1 NDM- and IMP-positive isolate (Table 2). Excluding isolates cocarrying an MBL, 230 isolates (18.2% of isolates that qualified for molecular characterization) were KPC positive, and 168 isolates (13.3%) were OXA-48-like positive (and MBL and KPC negative). ESBLs (in the absence of carbapenemase genes) were identified in 534 (42.2%) isolates that qualified for molecular characterization, with CTX-M-1-group-positive isolates accounting for 73.6% (393/534; 89.1% [350/393] of CTX-M-1 group-positive isolates carried CTX-M-15) of isolates with ESBLs. Acquired AmpC enzymes (in the absence of carbapenemase and ESBL genes) were uncommon among molecularly characterized *Enterobacterales* (*n *= 34; 2.7%).

**TABLE 2:** *In vitro* activity of cefepime-taniborbactam and comparator agents against 1,265 isolates of *Enterobacterales* with molecularly identified β-lactamase genotypes

Genotype (no. of isolates; % of total molecularly characterized isolates)	Antimicrobial agent	MIC (μg/mL)			MIC interpretation		
		MIC_50_	MIC_90_	MIC range	Susceptible (%)	Intermediate (%)	Resistant (%)
Carbapenemase positive (633; 50.0)^*c*^	Cefepime-taniborbactam^*a*^	1	16	0.016 to >16	94.6	NA^*b*^	5.4
	Ceftazidime-avibactam	4	>16	≤0.12 to >16	59.6	NA	40.4
	Ceftolozane-tazobactam	>8	>8	≤0.25 to >8	0.9	2.1	97.0
	Meropenem-vaborbactam	8	>16	≤0.06 to >16	45.2	7.0	47.9
	Piperacillin-tazobactam	>128	>128	≤4 to >128	0.5	2.2	97.3
							
ESBL positive (534; 42.2)^*d*^	Cefepime-taniborbactam	0.12	1	0.016 to >16	98.7	NA	1.3
	Ceftazidime-avibactam	0.25	1	≤0.12 to >16	98.5	NA	1.5
	Ceftolozane-tazobactam	1	>8	≤0.25 to >8	77.3	5.4	17.2
	Meropenem-vaborbactam	≤0.06	0.12	≤0.06–16	99.6	0.2	0.2
	Piperacillin-tazobactam	8	>128	≤4 to >128	74.3	10.5	15.2
							
CTX-M-1 positive (393; 31.1)	Cefepime-taniborbactam	0.12	1	0.016 to 8	100	NA	0
	Ceftazidime-avibactam	0.25	1	≤0.12 to >16	99.0	NA	1.0
	Ceftolozane-tazobactam	1	>8	≤0.25 to >8	78.6	5.1	16.3
	Meropenem-vaborbactam	≤0.06	0.12	≤0.06 to 8	99.7	0.3	0
	Piperacillin-tazobactam	8	>128	≤4 to >128	73.3	12.7	14.0
							
MBL positive (235; 18.6)^*e*^	Cefepime-taniborbactam	1	>16	0.03 to >16	86.4	NA	13.6
	Ceftazidime-avibactam	>16	>16	0.25 to >16	0.9	NA	99.2
	Ceftolozane-tazobactam	>8	>8	>8	0	0	100
	Meropenem-vaborbactam	>16	>16	0.25 to >16	8.9	8.1	83.0
	Piperacillin-tazobactam	>128	>128	≤4 to >128	1.3	3.0	95.7
							
KPC positive (230; 18.2)^*f*^	Cefepime-taniborbactam	0.5	4	0.016 to 8	100	NA	0
	Ceftazidime-avibactam	2	8	≤0.012 to >16	93.9	NA	6.1
	Ceftolozane-tazobactam	>8	>8	2 to >8	0.4	2.6	97.0
	Meropenem-vaborbactam	≤0.06	2	≤0.06 to >16	93.5	4.8	1.7
	Piperacillin-tazobactam	>128	>128	32 to >128	0	2.6	97.4
							
NDM positive (207; 16.4)^*g*^	Cefepime-taniborbactam	2	>16	0.12 to >16	84.6	NA	15.4
	Ceftazidime-avibactam	>16	>16	0.25 to >16	0.5	NA	99.5
	Ceftolozane-tazobactam	>8	>8	>8	0	0	100
	Meropenem-vaborbactam	>16	>16	4 to >16	3.4	7.7	88.9
	Piperacillin-tazobactam	>128	>128	32 to >128	0	1.9	98.1
							
OXA-48 group positive (168; 13.3)^*h*^	Cefepime-taniborbactam	2	4	0.03 to >16	98.8	NA	1.2
	Ceftazidime-avibactam	1	2	≤0.12 to >16	94.6	NA	5.4
	Ceftolozane-tazobactam	>8	>8	≤0.25 to >8	3.0	4.2	92.9
	Meropenem-vaborbactam	16	>16	0.25 to >16	29.8	8.3	61.9
	Piperacillin-tazobactam	>128	>128	64 to >128	0	0.6	99.4
							
CTX-M-9 positive (73; 5.8)	Cefepime-taniborbactam	0.06	0.12	0.016 to 4	100	NA	0
	Ceftazidime-avibactam	≤0.12	0.25	≤0.12 to 0.5	100	NA	0
	Ceftolozane-tazobactam	0.5	1	≤0.25 to 2	100	0	0
	Meropenem-vaborbactam	≤0.06	≤0.06	≤0.06 to 0.12	100	0	0
	Piperacillin-tazobactam	≤4	8	≤4 to 16	100	0	0
							
Acquired AmpC positive (34; 2.7)^*i*^	Cefepime-taniborbactam	0.06	2	0.016 to 2	100	NA	0
	Ceftazidime-avibactam	0.25	2	≤0.12 to >16	94.1	NA	5.9
	Ceftolozane-tazobactam	2	>8	≤0.25 to >8	58.8	8.8	32.4
	Meropenem-vaborbactam	≤0.06	0.25	≤0.06 to 4	100	0	0
	Piperacillin-tazobactam	8	>128	≤4 to >128	79.4	5.9	14.7
							
SHV ESBL positive (23; 1.8)	Cefepime-taniborbactam	0.06	0.25	0.03 to 1	100	NA	0
	Ceftazidime-avibactam	0.5	2	≤0.12 to 4	100	NA	0
	Ceftolozane-tazobactam	1	>8	≤0.25 to >8	73.9	4.4	21.7
	Meropenem-vaborbactam	≤0.06	0.12	≤0.06 to 0.12	100	0	0
	Piperacillin-tazobactam	8	>128	≤4 to >128	73.9	4.4	21.7
							
VIM positive (22; 1.7)^*j*^	Cefepime-taniborbactam	1	8	0.03 to 16	100	NA	0
	Ceftazidime-avibactam	>16	>16	4 to >16	4.6	NA	95.5
	Ceftolozane-tazobactam	>8	>8	>8	0	0	100
	Meropenem-vaborbactam	4	>16	0.5 to >16	50.0	4.6	45.5
	Piperacillin-tazobactam	>128	>128	32 to >128	0	4.6	95.5

a For comparative purposes only, percent susceptible and percent resistant for cefepime-taniborbactam correspond to the percentage of isolates inhibited at ≤16 μg/mL and ≥32 μg/mL, respectively.

b NA, not applicable.

c Includes isolates with MBLs and serine carbapenemases; isolates could also possess OSBLs (original spectrum β-lactamases, e.g., TEM-1, SHV-1, etc.), ESBLs, or AmpC-type enzymes.

d Isolates could also possess AmpC-type enzymes, or OSBLs, but no carbapenemases.

e Includes isolates that possess IMP (*n *= 5), NDM (*n *= 207), and VIM (*n *= 22). One isolate possessed both NDM and IMP.

f Isolates could also possess the OXA-48 group, ESBLs, AmpC-type enzymes, OSBLs but not MBLs.

g Isolates could also possess serine carbapenemases, ESBLs, AmpCs, and/or OSBLs, but no other MBLs.

h Isolates could also possess ESBLs, AmpC-type enzymes, or OSBLs, but no other carbapenemases.

i Isolates could also possess OSBLs, but no ESBLs or carbapenemases.

j Isolates could also possess serine carbapenemases, ESBLs, AmpCs, and/or OSBLs, but no other MBLs.

**FIG 1:**
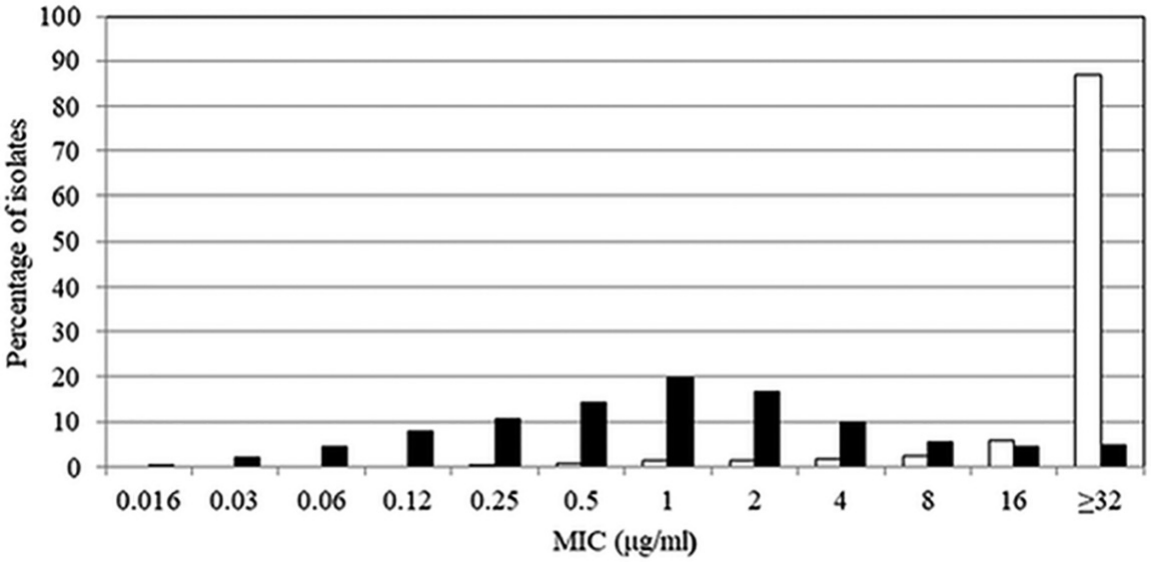
Cefepime (white bars) and cefepime-taniborbactam (black bars) MIC frequency distributions for 627 isolates of carbapenemase-positive *Enterobacterales*. The 627 isolates of carbapenemase-positive *Enterobacterales* included 230 KPC-positive, 207 NDM-positive, 168 OXA-48 group-positive, and 22 VIM-positive isolates (note: several isolates carried multiple carbapenemases). IMP-positive isolates (*n *= 6) were excluded from the data set as IMP is outside the spectrum of taniborbactam inhibition.

Cefepime-taniborbactam (MIC_50_, 1 μg/mL; MIC_90_, 16 μg/mL), ceftazidime-avibactam (MIC_50_, 4 μg/mL; MIC_90_, >16 μg/mL), and meropenem-vaborbactam (MIC_50_, 8 μg/mL; MIC_90_, 16 μg/mL) inhibited 94.6%, 59.6%, and 45.2%, respectively, of 633 carbapenemase-positive isolates of *Enterobacterales* (Table 2). Ceftolozane-tazobactam and piperacillin-tazobactam were inactive against carbapenemase-positive *Enterobacterales*. Cefepime-taniborbactam inhibited 86.4% of MBL-positive isolates overall, including 100% of VIM-positive and 84.6% of NDM-positive isolates. Ceftazidime-avibactam and meropenem-vaborbactam were inactive against MBL-positive isolates. Cefepime-taniborbactam (100% inhibited at ≤16 μg/mL), ceftazidime-avibactam (93.9% susceptible), and meropenem-vaborbactam (93.5% susceptible) were active against *Enterobacterales* carrying KPCs. Cefepime-taniborbactam (98.8% inhibited at ≤16 μg/mL) and ceftazidime-avibactam (94.6% susceptible) were active against *Enterobacterales* carrying OXA-48-like enzymes, while meropenem-vaborbactam was poorly active (29.8% susceptible). Cefepime-taniborbactam also inhibited 98.7% of ESBL-positive isolates (MIC_90_, 1 μg/mL) and 100% of acquired AmpC-positive isolates (MIC_90_, 2 μg/mL) at ≤16 μg/mL. The MIC distributions for cefepime-taniborbactam and cefepime against the 627 carbapenemase-positive isolates of *Enterobacterales* (excluding the six IMP-positive isolates) are depicted in Fig. 1. Table S3 depicts the cefepime-taniborbactam MIC distribution for the 627 isolates of carbapenemase-positive *Enterobacterales* stratified by carbapenemase type and susceptibility to cefepime.

### *Enterobacterales*: characterization of isolates with elevated cefepime-taniborbactam MICs.

The 74 isolates of *Enterobacterales* with elevated cefepime-taniborbactam MICs (≥16 μg/mL) were 41 K. pneumoniae, 24 E. coli, 3 *P. rettgeri*, 3 S. marcescens, 2 E. cloacae, and 1 Citrobacter freundii. Each isolate carried one or more β-lactamase genes, and 83.8% of isolates carried one or more carbapenemase genes.

Putative explanations for elevated cefepime-taniborbactam MIC values could be deduced for all 41 K. pneumoniae isolates. Two isolates carried IMP-8. Of the remaining 39 isolates, 32 harbored NDM (not sufficient on its own to confer cefepime-taniborbactam MICs of ≥32 μg/mL) that in combination with other resistance factors such as porin mutations, may account for the observed decrease in susceptibility. One isolate had an *ftsI* gene encoding PBP3 (the primary target of cefepime) with a four-amino acid insertion (TVPY) at position 334 and also possessed NDM-5, OXA-181, and CTX-M-15 (all within the spectrum of taniborbactam). While PBP3 insertions at position 334 have been described in E. coli, they have not been previously reported in Klebsiella spp. (17). Of the 41 isolates, 38 (92.7%) had a disruption or alteration likely affecting drug entry. A total of 30 isolates possessed a disruption in *ompK35* (OmpF), and 38 isolates had a disruption in *ompK36* (OmpC) in regions of each porin known to affect permeability. Two isolates showed a disruption in *ramR*, and another isolate carried for a 15-amino acid deletion in RamR, a negative regulator of RamA, itself an enhancer of AcrAB efflux pump expression (and multidrug resistance) in K. pneumoniae (18).

**FIG 2:**
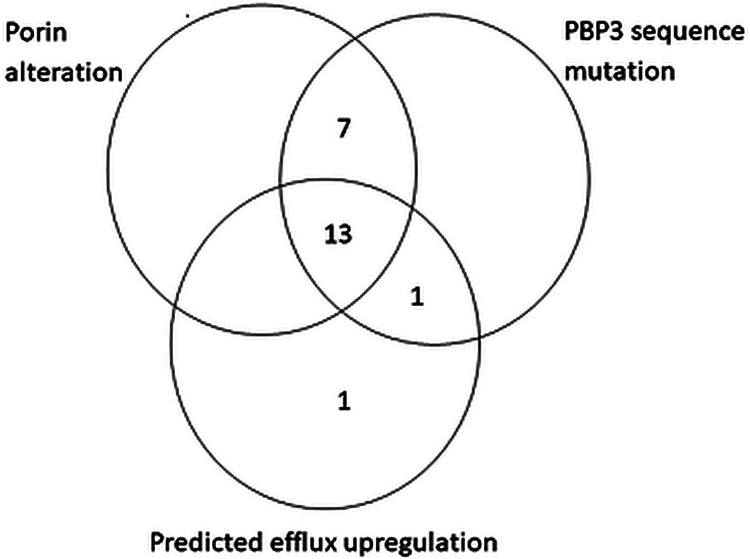
Occurrence and cooccurrence of mechanisms of antimicrobial resistance identified in 22/24 isolates of E. coli with cefepime-taniborbactam MICs of ≥16 μg/mL. Major porin genes, *ompC* and *ompF*, were screened for alterations that code for a truncated, presumably nonfunctional protein. PBP3 sequence mutation includes *ftsI* sequences predicted to code insertions known to reduce cefepime binding. Predicted efflux upregulation includes genetic changes that likely enhance drug extrusion.

Putative resistance factors contributing to elevated cefepime-taniborbactam MIC values were identified in 22 of the 24 (91.7%) E. coli isolates. Of the 24 isolates, 21 (87.5%) showed 4 amino acid insertions (YRIN [*n *= 13] or YRIK [*n *= 8]) at position 333 in PBP3 that are known to negatively impact the accessibility of aztreonam and cephalosporins to the transpeptidase binding site (19). The three remaining isolates exhibited wild-type *ftsI*. A total of 17 isolates carried NDM (14 from India, 2 from Russia, 1 from Mexico). Each of the 17 NDM-positive isolates also possessed one of the aforementioned PBP3 insertions. Of the 24 isolates, 20 (83.3%) displayed an alteration in OmpC and/or OmpF, likely resulting in reduced permeability. Alterations suggesting a nonfunctional protein were observed for *marR* (AcrAB efflux pump regulatory gene) in one isolate and in *acrR* (another AcrAB efflux pump regulatory gene) in the same isolate and 14 others. Fig. 2 summarizes the overlap of mutations in porin genes, *ftsI*, and efflux regulatory genes in *Enterobacterales* with elevated cefepime-taniborbactam MICs.

Among the remaining *Enterobacterales* with elevated cefepime-taniborbactam MIC values, the single C. freundii isolate carried an IMP-8 gene, and both E. cloacae isolates also carried IMP (one isolate carried IMP-4 and one isolate carried IMP-8). Among the three *P. rettgeri* isolates, one harbored IMP-27. The remaining two isolates carried NDM-1, CMY-4, and OXA-181 (a member of the OXA-48 group of carbapenemases); one of the two also carried the ESBL, VEB-1 (all within the spectrum of taniborbactam). The three S. marcescens isolates carried CTX-M-15 and OXA-1. Reduced susceptibility to cefepime has been linked to OXA-1 carriage in *Enterobacterales* (20).

In a supplemental study of 80 E. coli isolates with cefepime-taniborbactam MICs of ≤8 μg/mL, we observed 17 isolates with YRIN or YRIK insertions at position 333 of PBP3. PBP3 insertions in isolates with an acquired β-lactamase, particularly NDM, may be sufficient to increase the cefepime-taniborbactam MIC to 16 μg/mL or higher.

### P. aeruginosa: all isolates and those with antimicrobial-resistant phenotypes.

The 4,619 P. aeruginosa isolates tested were 30.6% imipenem resistant, 21.2% ciprofloxacin resistant, 20.5% meropenem resistant, 18.4% ceftazidime resistant, 15.0% piperacillin-tazobactam resistant, 12.2% gentamicin resistant, and 11.7% cefepime resistant (Table S4). MDR and DTR phenotypes were identified in 17.5% and 10.2% of P. aeruginosa isolates, respectively (Table 3).

**TABLE 3:** *In vitro* activity of cefepime-taniborbactam and comparator agents against isolates of P. aeruginosa with antimicrobial-resistant phenotypes

Phenotype (no. of isolates; % of total)	Antimicrobial agent	MIC (μg/mL)			MIC interpretation		
		MIC_50_	MIC_90_	MIC range	Susceptible (%)	Intermediate (%)	Resistant (%)
All isolates (4,619; 100)	Cefepime-taniborbactam^*a*^	2	8	≤0.06 to >32	97.4	NA^*b*^	2.6
	Ceftazidime-avibactam	2	8	≤0.25 to >16	90.5	NA	9.6
	Ceftolozane-tazobactam	1	8	≤0.12 to >16	88.8	2.6	8.7
	Meropenem-vaborbactam	0.5	16	≤0.06 to >16	86.6	NA	13.4
	Piperacillin-tazobactam	8	>128	≤0.5 to >128	70.8	14.2	15.0
							
Imipenem resistant (1,415; 30.6)	Cefepime-taniborbactam	8	16	0.25 to >32	92.4	NA	7.6
	Ceftazidime-avibactam	4	>16	≤0.25 to >16	71.7	NA	28.3
	Ceftolozane-tazobactam	2	>16	0.25 to >16	68.1	5.6	26.4
	Meropenem-vaborbactam	8	>16	≤0.06 to >16	57.0	NA	43.0
	Piperacillin-tazobactam	32	>128	≤0.25 to >128	39.6	27.3	33.0
							
Meropenem resistant (948; 20.5)	Cefepime-taniborbactam	8	32	0.5 to >32	89.0	NA	11.0
	Ceftazidime-avibactam	8	>16	0.5 to >16	59.8	NA	40.2
	Ceftolozane-tazobactam	4	>16	0.5 to >16	55.6	7.4	37.0
	Meropenem-vaborbactam	16	>16	0.5 to >16	34.7	NA	65.3
	Piperacillin-tazobactam	64	>128	1 to >128	20.2	36.5	43.4
							
MDR phenotype (807; 17.5)^*c*^	Cefepime-taniborbactam	8	>32	0.5 to >32	85.7	NA	14.3
	Ceftazidime-avibactam	16	>16	0.5 to >16	48.7	NA	51.3
	Ceftolozane-tazobactam	8	>16	0.5 to >16	41.1	10.0	48.8
	Meropenem-vaborbactam	16	>16	≤0.06 to >16	40.1	NA	59.9
	Piperacillin-tazobactam	128	>128	1 to >128	7.4	29.6	62.9
							
Piperacillin-tazobactam resistant (692; 15.0)	Cefepime-taniborbactam	8	>32	0.5 to >32	87.7	NA	12.3
	Ceftazidime-avibactam	8	>16	0.5 to >16	59.8	NA	40.2
	Ceftolozane-tazobactam	4	>16	0.5 to >16	54.9	14.9	30.2
	Meropenem-vaborbactam	8	>16	≤0.06 to >16	57.1	NA	42.9
	Piperacillin-tazobactam	>128	>128	128 to >128	0	0	100
							
Meropenem-vaborbactam resistant (619; 13.4)	Cefepime-taniborbactam	8	>32	1 to >32	85.0	NA	15.0
	Ceftazidime-avibactam	16	>16	1 to >16	45.6	NA	54.4
	Ceftolozane-tazobactam	8	>16	0.5 to >16	43.3	7.1	49.6
	Meropenem-vaborbactam	>16	>16	16 to >16	0	NA	100
	Piperacillin-tazobactam	64	>128	4 to >128	9.7	42.3	48.0
							
Cefepime resistant (538; 11.6)	Cefepime-taniborbactam	8	>32	1 to >32	78.3	NA	21.7
	Ceftazidime-avibactam	16	>16	0.5 to >16	35.9	NA	64.1
	Ceftolozane-tazobactam	>16	>16	1 to >16	25.8	17.5	56.7
	Meropenem-vaborbactam	16	>16	≤0.06 to >16	41.1	NA	58.9
	Piperacillin-tazobactam	>128	>128	2 to >128	3.7	23.6	72.7
							
DTR phenotype (470; 10.2)^*d*^	Cefepime-taniborbactam	8	>32	1 to >32	79.4	NA	20.6
	Ceftazidime-avibactam	>16	>16	0.5 to >16	29.1	NA	70.9
	Ceftolozane-tazobactam	>16	>16	1 to >16	22.6	9.1	68.3
	Meropenem-vaborbactam	>16	>16	0.5 to >16	23.2	NA	76.8
	Piperacillin-tazobactam	128	>128	32 to >128	0	36.0	64.0
							
Ceftazidime-avibactam resistant (441; 9.5%)	Cefepime-taniborbactam	8	>32	1 to >32	79.1	NA	20.9
	Ceftazidime-avibactam	>16	>16	16 to >16	0	NA	100
	Ceftolozane-tazobactam	>16	>16	2 to >16	14.3	11.6	74.2
	Meropenem-vaborbactam	>16	>16	0.25 to >16	23.6	NA	76.4
	Piperacillin-tazobactam	>128	>128	2 to >128	4.1	32.9	63.0
							
Ceftolozane-tazobactam resistant (401; 8.7)	Cefepime-taniborbactam	8	>32	1 to >32	78.1	NA	21.9
	Ceftazidime-avibactam	>16	>16	1 to >16	18.5	NA	81.6
	Ceftolozane-tazobactam	>16	>16	16 to >16	0	0	100
	Meropenem-vaborbactam	>16	>16	≤0.06 to >16	23.4	NA	76.6
	Piperacillin-tazobactam	128	>128	2 to >128	6.5	41.4	52.1

a For comparative purposes only, percent susceptible and percent resistant for cefepime-taniborbactam correspond to the percentage of isolates inhibited at ≤16 μg/mL and ≥32 μg/mL, respectively.

b NA, not applicable.

^c^An MDR phenotype was assigned to isolates resistant to at least one agent from ≥3 of the following antimicrobial agent classes: aminoglycosides (gentamicin), β-lactam combination agents (piperacillin-tazobactam, ceftazidime-avibactam, ceftolozane-tazobactam, meropenem-vaborbactam), carbapenems (meropenem or imipenem), cephems (ceftazidime, cefepime), and fluoroquinolones (levofloxacin or ciprofloxacin).

d DTR isolates were identified using the definition of Kadri et al. (42) as isolates intermediate or resistant to fluoroquinolones (ciprofloxacin) and all β-lactams, including carbapenems and piperacillin-tazobactam but excluding ceftazidime-avibactam, ceftolozane-tazobactam, and meropenem-vaborbactam.

Against all P. aeruginosa isolates, the cefepime-taniborbactam MIC_50_ and MIC_90_ were 2 and 8 μg/mL, respectively; 97.4% of isolates were inhibited at ≤16 μg/mL (Table 3). At a cefepime-taniborbactam concentration of ≤8 μg/mL, 94.2% of all P. aeruginosa isolates were inhibited (data not shown). The addition of taniborbactam to cefepime reduced the MIC_90_ value by 4-fold from 32 μg/mL to 8 μg/mL (Table S4). The most active comparator against P. aeruginosa overall was ceftazidime-avibactam (90.5% susceptible); all other comparators inhibited <90% of isolates, including ceftolozane-tazobactam (88.8% susceptible), meropenem-vaborbactam (86.6% susceptible [by EUCAST breakpoints]), meropenem (73.5% susceptible), and piperacillin-tazobactam (70.8% susceptible).

At a concentration of ≤16 μg/mL, cefepime-taniborbactam inhibited 92.4% of imipenem-resistant, 89.0% of meropenem-resistant, 85.7% of MDR, 87.7% of piperacillin-tazobactam-resistant, 85.0% of meropenem-vaborbactam-resistant, 78.3% of cefepime-resistant, 79.4% of DTR, 79.1% of ceftazidime-avibactam-resistant, and 78.1% of ceftolozane-tazobactam-resistant P. aeruginosa isolates (Table 3). At ≤16 μg/mL, cefepime-taniborbactam inhibited a greater percentage of isolates with each of the nine resistance phenotypes studied than all tested comparators.

### P. aeruginosa: isolates with antimicrobial-resistant genotypes.

Among all P. aeruginosa isolates that qualified for molecular characterization (*n *= 1,110), 249 (22.4%) were carbapenemase positive, including 209 that were MBL positive. The MBL-positive isolates included 159 (76.1%) VIM-positive, 28 IMP-positive (13.4%), and 16 (7.7%) NDM-positive isolates. Three isolates cocarried IMP and VIM, two cocarried IMP and DIM, and one cocarried NDM and DIM. Additional molecularly characterized P. aeruginosa isolates carrying carbapenemases included seven with KPC and 33 carrying GES variants (19 isolates with GES-5, 6 with GES-19-20, 6 with GES-20, and 2 with GES-6) with reported carbapenemase activity.

**FIG 3:**
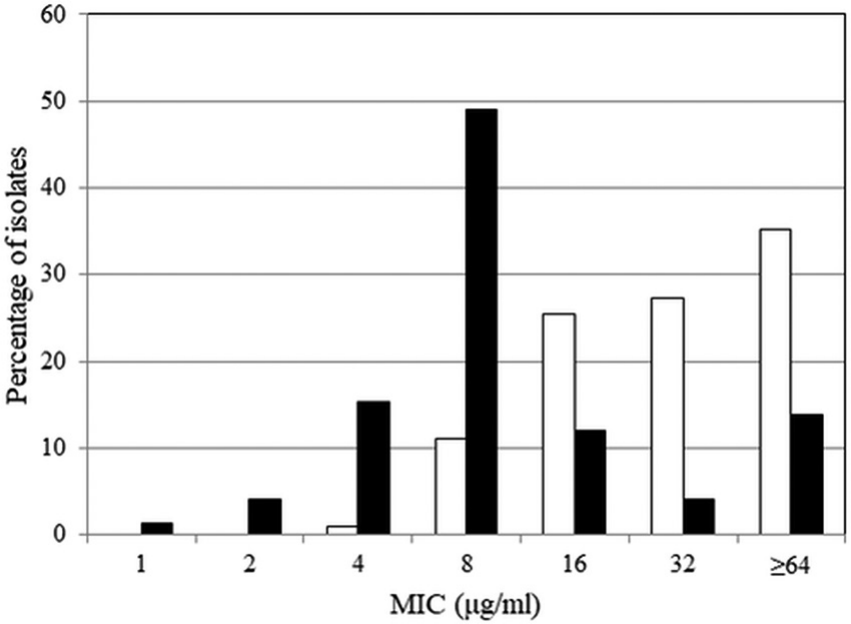
Cefepime (white bars) and cefepime-taniborbactam (black bars) MIC frequency distributions for 216 isolates of carbapenemase-positive P. aeruginosa. The 216 isolates of carbapenemase-positive P. aeruginosa included 159 VIM-positive, 33 GES-positive (GES-5, GES-6, GES-19/20, GES-20 [GES enzymes with reported carbapenemase activity]), and 17 NDM-positive isolates (note: several isolates carried multiple carbapenemases). IMP-positive isolates (*n *= 33) were excluded from the data set as IMP is outside the spectrum of taniborbactam inhibition.

Cefepime-taniborbactam was the most active agent against carbapenemase-producing subsets of P. aeruginosa, inhibiting 71.5% of all carbapenemase-positive isolates (MIC_50_, 8 μg/mL; MIC_90_, >32 μg/mL), 66.5% of all MBL-positive isolates (MIC_50_, 8 μg/mL; MIC_90_, >32 μg/mL), 87.4% of VIM-positive isolates (MIC_50_, 8 μg/mL; MIC_90_, 32 μg/mL), and 100% of GES carbapenemase-positive isolates (MIC_50_, 8 μg/mL; MIC_90_, 16 μg/mL) at ≤16 μg/mL (Table 4). Ceftazidime-avibactam, meropenem-vaborbactam, ceftolozane-tazobactam, and piperacillin-tazobactam were inactive against carbapenemase-positive P. aeruginosa with the notable exception that 75.8% of GES carbapenemase-positive isolates (*n *= 33) were susceptible to ceftazidime-avibactam. The MIC distributions for cefepime-taniborbactam and cefepime against the 216 carbapenemase-positive isolates of P. aeruginosa (excluding the 33 IMP-positive isolates) are depicted in Fig. 3. Table S5 depicts the cefepime-taniborbactam MIC distribution for the 216 carbapenemase-positive P. aeruginosa isolates stratified by carbapenemase type and susceptibility to cefepime.

**TABLE 4:** *In vitro* activity of cefepime-taniborbactam and comparator agents against 1,110 isolates of P. aeruginosa with molecularly identified β-lactamase genotypes

Genotype (no. of isolates; % of total molecularly characterized isolates)	Antimicrobial agent	MIC (μg/mL)			MIC interpretation		
		MIC_50_	MIC_90_	MIC range	Susceptible (%)	Intermediate (%)	Resistant (%)
Carbapenemase positive (249; 22.4)^*a*^	Cefepime-taniborbactam^*b*^	8	>32	1 to >32	71.5	NA^*c*^	28.5
	Ceftazidime-avibactam	>16	>16	2 to >16	14.9	NA	85.1
	Ceftolozane-tazobactam	>16	>16	1 to >16	1.2	2.4	96.4
	Meropenem-vaborbactam	>16	>16	1 to >16	7.6	NA	92.4
	Piperacillin-tazobactam	64	>128	2 to >128	3.2	49.4	47.4
							
MBL positive (209; 18.8)^*d*^	Cefepime-taniborbactam	8	>32	1 to >32	66.5	NA	33.5
	Ceftazidime-avibactam	>16	>16	4 to >16	2.9	NA	97.1
	Ceftolozane-tazobactam	>16	>16	1 to >16	1.0	0	99.0
	Meropenem-vaborbactam	>16	>16	4 to >16	7.7	NA	92.3
	Piperacillin-tazobactam	64	>128	2 to >128	3.4	49.3	47.4
							
VIM positive (159; 14.3)^*e*^	Cefepime-taniborbactam	8	32	1 to >32	87.4	NA	12.6
	Ceftazidime-avibactam	>16	>16	4 to >16	3.8	NA	96.2
	Ceftolozane-tazobactam	>16	>16	1 to >16	1.3	0	98.7
	Meropenem-vaborbactam	>16	>16	4 to >16	6.9	NA	93.1
	Piperacillin-tazobactam	64	>128	16 to >128	2.5	56.6	40.9
							
GES positive (51; 4.6)^*f*^	Cefepime-taniborbactam	8	16	1 to >32	98.0	NA	2.0
	Ceftazidime-avibactam	8	>16	2 to >16	54.9	NA	45.1
	Ceftolozane-tazobactam	>16	>16	8 to >16	0	11.8	88.2
	Meropenem-vaborbactam	>16	>16	0.25 to >16	21.6	NA	78.4
	Piperacillin-tazobactam	64	>128	16 to >128	5.9	54.9	39.24
							
VEB positive (21; 1.9)	Cefepime-taniborbactam	8	8	4 to 8	100	NA	0
	Ceftazidime-avibactam	>16	>16	16 to >16	0		100
	Ceftolozane-tazobactam	>16	>16	>16	0	0	100
	Meropenem-vaborbactam	>16	>16	16 to >16	0		100
	Piperacillin-tazobactam	128	>128	32 to >128	0	42.9	57.1

a Includes isolates harboring GES-2, GES-5, GES-6, and GES-20 (variants with reported carbapenemase activity).

b For comparative purposes only, percent susceptible and percent resistant for cefepime-taniborbactam correspond to the percentage of isolates inhibited at ≤16 μg/mL and ≥32 μg/mL, respectively.

c NA, not applicable.

d Includes isolates harboring VIM (*n *= 159), IMP (*n *= 28), and NDM (*n *= 16). Two isolates cocarried IMP and DIM, three isolates cocarried IMP and VIM, and one isolate cocarried NDM and DIM.

e Isolates could also possess serine carbapenemases, ESBLs, AmpCs, and/or OSBLs, but no other MBLs.

f The 51 GES-positive isolates comprised 33 isolates with a GES carbapenemase and 18 isolates with a GES ESBL.

### P. aeruginosa: characterization of isolates with elevated cefepime-taniborbactam MICs.

**FIG 4:**
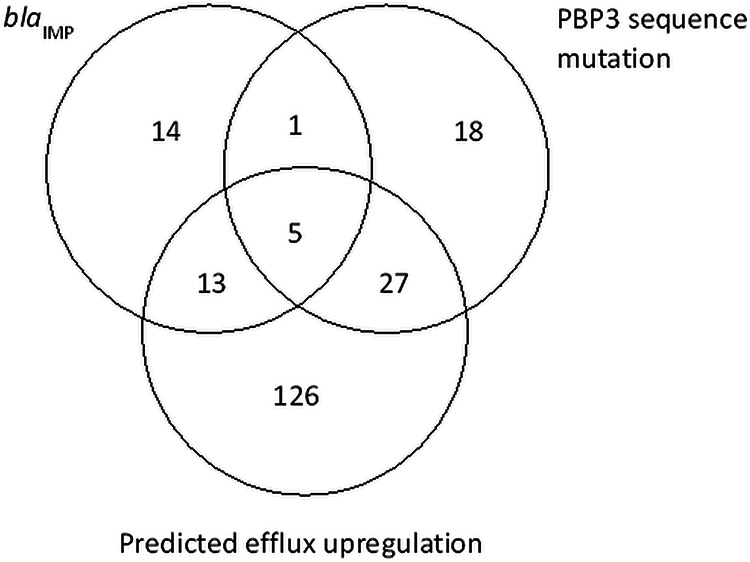
Occurrence and cooccurrence of mechanisms of antimicrobial resistance identified in 204/267 isolates of P. aeruginosa with cefepime-taniborbactam MICs of ≥16 μg/mL. Resistance factors include the presence of IMP family MBLs, mutation in the *ftsI* sequence predicted to code for PBP3 variants with reduced cefepime binding, and predicted efflux upregulation that includes genetic changes that likely enhance drug extrusion.

Of the 267 P. aeruginosa isolates with cefepime-taniborbactam MICs of ≥16 μg/mL, 33 (12.4%) carried IMP. A total of 109 isolates (40.8%) had a PBP3 amino acid sequence that differed from the wild-type reference; of these, 51 isolates showed substitutions implicated in elevated resistance to cephalosporins, including cefepime, such as G63D, G216S, A244T, R504C, I524T, P527S, G531D, and F533L (21–23). Alterations in, or absence of, genes coding for negative regulators of efflux systems (*esrC*, *mexR*, *mexS*, *mexT*, *mexZ*, *nalC*, *nalD*, *nfxB*) were identified in 64.0% (171/267) of isolates. Loss-of-function mutations in these loci are expected to result in upregulation of multidrug efflux pumps in P. aeruginosa. Other mutations associated with increased efflux in *nfxB* (A30T, R21H, R82L, G180S) (24) were also observed in 6 additional isolates; 1 isolate had a 23-amino acid deletion in MexR. The *oprD* gene contained a genetic alteration (*n *= 190) or lacked the gene (*n *= 2) in 71.9% (192/267) of isolates. However, OprD deficiency is associated primarily with resistance to carbapenems, not to cefepime. Of the 267 isolates, 38 (14.2%) exhibited a genetic disruption in one or more of the regulatory genes (*ampD*, *dacB*, *mpl*, *ampR*), putatively resulting in overexpression of Pseudomonas-derived cephalosporinase (PDC), the intrinsic AmpC in P. aeruginosa (21, 23). Additionally, 19 isolates were found to possess amino acid substitutions in AmpD (H77Y, R82C, F89S, A96T, T139A, or P162L) or AmpR (D135N or G154R) that have been reported to lead to AmpC overexpression (25–28). Cefepime is only a weak inducer of AmpC and withstands hydrolysis by AmpC due to formation of a stable acyl-enzyme complex (29). However, it is possible that cefepime activity may be reduced against isolates that overexpress AmpC. Figure 4 summarizes the overlap among the presence of IMP β-lactamases, *ftsI* mutations that code for PBP3 variants expected to result in elevated MICs for cefepime and other cephalosporins, and efflux-related genes with variations that potentially enhance efflux activity in P. aeruginosa isolates with elevated cefepime-taniborbactam MIC values.

### Enterobacterales and P. aeruginosa: carbapenem-resistant, carbapenemase-negative isolates.

Against CRE, cefepime-taniborbactam inhibited 37 to >90% more isolates with carbapenemases (*n *= 596) and 5 to >90% more isolates without carbapenemases (*n *= 41) than the other β-lactam/β-lactamase inhibitor combinations tested (Table 5). Similarly, against CRPA, cefepime-taniborbactam inhibited 57 to 70% more isolates with carbapenemases (*n *= 249) and 19 to 69% more isolates without carbapenemases (*n *= 753) than the other β-lactam/β-lactamase inhibitor combinations tested.

**TABLE 5:** *In vitro* activity of cefepime-taniborbactam and comparator agents against carbapenem-resistant isolates of *Enterobacterales* and P. aeruginosa stratified by the presence and absence of molecularly identified carbapenemases

Antimicrobial agent	No. (%) of susceptible isolates of:			
	Carbapenem-resistant *Enterobacterales* (CRE)		Carbapenem-resistant P. aeruginosa (CRPA)	
	Without carbapenemase (*n *= 41)	With carbapenemase (*n *= 596)	Without carbapenemase (*n *= 753)	With carbapenemase (*n *= 249)
Cefepime-taniborbactam^*a*^	39 (95.1)	563 (94.5)	714 (94.8)	177 (71.1)
Ceftazidime-avibactam	31 (79.5)	345 (57.9)	574 (76.2)	36 (14.5)
Ceftolozane-tazobactam	2 (4.9)	5 (0.8)	565 (75.0)	2 (0.8)
Meropenem-vaborbactam	37 (90.2)	249 (41.8)	365 (48.5)	18 (7.2)
Piperacillin-tazobactam	1 (2.4)	0 (0)	198 (26.3)	8 (3.2)

a For comparative purposes only, percent susceptible and percent resistant for cefepime-taniborbactam correspond to the percentage of isolates inhibited at ≤16 μg/mL and ≥32 μg/mL, respectively.

## DISCUSSION

Against a prevalence-based collection of 18,350 clinical isolates of *Enterobacterales* and P. aeruginosa, cefepime-taniborbactam at ≤16 μg/mL inhibited greater percentages of *Enterobacterales* and P. aeruginosa isolates with important resistance phenotypes and genotypes than ceftazidime-avibactam, meropenem-vaborbactam, ceftolozane-tazobactam, and piperacillin-tazobactam.

In the current study, cefepime-taniborbactam inhibited 86.4% of all MBL-positive *Enterobacterales* isolates, including all VIM-positive isolates and 84.6% of NDM-positive isolates. A previous smaller study of 42 VIM-positive (MIC_90_, 4 μg/mL) and 10 NDM-positive (MIC_90_, 16 μg/mL) isolates of *Enterobacterales* reported similar results (15). Only 2 of the 52 isolates in that study yielded cefepime-taniborbactam MICs of >16 μg/mL: 1 NDM producer with an insertion in PBP3 and 1 VIM-1 producer with nonfunctional OmpK35. Other studies have reported cefepime-taniborbactam MICs of ≤4 μg/mL for all NDM-positive (*n *= 13) and VIM-positive (*n *= 11) *Enterobacterales* isolates tested (14) and reported that 92% (92/100) of MBL-positive K. pneumoniae isolates were inhibited by cefepime-taniborbactam at ≤16 μg/mL (13). Wang et al. tested 87 NDM-positive *Enterobacterales* isolates from China and reported that 67% of isolates had cefepime-taniborbactam MICs of ≤16 μg/mL (16). In that study, *bla*_NDM-5_-positive E. coli (76.1%, 35/46) were the majority of the isolates with elevated cefepime-taniborbactam MICs, and almost all isolates of this subset had a YRIN or INYR insertion in PBP3, suggesting that PBP3 insertions were associated with elevated cefepime-taniborbactam MICs, as taniborbactam is known to inhibit NDM-5 (9). Canadian investigators identified two isolates of E. coli with cefepime-taniborbactam MICs of >16 μg/mL (32 μg/mL) among a set of 179 ertapenem-nonsusceptible isolates from over a decade of nationwide resistance surveillance; 1 possessed NDM-5, OXA-181 and TEM-1B, an OmpC alteration and a YRIN insertion in PBP3, while the second contained CTX-M-71, a truncated OmpF and a large alteration in OmpC (11). A study of *Enterobacterales* with cefepime-taniborbactam MICs of >8 μg/mL noted no universal resistance mechanism across the isolates tested but, rather, combinations of carbapenemases (e.g., NDM-5, NDM-7) with PBP3 insertions and/or porin changes (10).

The potent activity of cefepime-taniborbactam against KPC-, OXA-48-like-, ESBL-, and AmpC-positive *Enterobacterales* was observed in earlier, limited studies focusing on challenge sets of isolates (9–11, 15, 16). A study of 247 carbapenemase-producing *Enterobacterales* from Spain reported cefepime-taniborbactam MICs of ≤16 μg/mL for 98.5% (199/202) of serine carbapenemase-positive and 93.3% (42/45) of MBL-positive isolates (12).

In the current study, cefepime-taniborbactam was the most active agent tested against carbapenemase-positive isolates of P. aeruginosa, particularly VIM-positive isolates (87.4% inhibited at ≤16 μg/mL), whereas approved β-lactam/β-lactamase inhibitor combinations were inactive against this challenging subset. A previous study tested 100 MBL-positive P. aeruginosa and showed an MIC_90_ of 32 μg/mL for cefepime-taniborbactam, with 88.0% of isolates inhibited at ≤16 μg/mL (13). A second study tested 22 CRPA (3 isolates were IMP positive) and reported an MIC_90_ of 32 μg/mL, with 86.4% (18/22) of isolates having cefepime-taniborbactam MICs of ≤16 μg/mL (16). A third study reported that 81.1% of 122 meropenem-resistant Pseudomonas spp. isolates had cefepime-taniborbactam MICs of ≤16 μg/mL (12). In that study, all 30 isolates with GES serine carbapenemase (with or without KPC) and 81.6% (40/49) of VIM-positive isolates (with or without additional β-lactamases) had cefepime-taniborbactam MICs of ≤16 μg/mL (12). Cefepime-taniborbactam MICs were higher (MIC_90_, 32 μg/mL) in the 35.2% (43/122) of isolates with non-carbapenemase resistance mechanisms, with 67.4% (29/43) of isolates having cefepime-taniborbactam MICs of ≤16 μg/mL (12). That value is less than the 94.8% of 753 carbapenem-resistant, carbapenemase-negative isolates inhibited by ≤16 μg/mL cefepime-taniborbactam in the current study and may be due to differences in the epidemiology of carbapenem resistance or clonal representation between studies.

Our study identified the presence of IMP in isolates with elevated cefepime-taniborbactam MICs. Although quite rare in most regions (4), this enzyme is outside the inhibitory spectrum of taniborbactam (9, 10). Rare isolates of E. coli demonstrated 4-amino acid insertions in PBP3, the cefepime target, typically in the setting of ESBL and NDM production, while P. aeruginosa infrequently showed single-amino acid substitutions in PBP3, often in the setting of efflux derepression. Isolates with PBP3 mutations were largely from India and Russia. Permeability (porin) defects in *Enterobacterales* in the setting of ESBL, OXA, and MBL production were also identified in isolates with elevated cefepime-taniborbactam MICs. Derepression of efflux (notably in E. coli, K. pneumoniae, and P. aeruginosa) decreases the activity of β-lactams and most other classes of agents; mutations suggestive of enhancement of multiple efflux mechanisms were present in several isolates of P. aeruginosa with elevated cefepime-taniborbactam MICs. While of concern, changes in permeability and efflux are not horizontally transferred. Resistance was likely multifactorial in many isolates. Although the mechanisms of ceftazidime-avibactam and ceftolozane-tazobactam resistance in the present study have not been characterized, cefepime-taniborbactam retains activity against ceftazidime-avibactam-resistant and ceftolozane-tazobactam-resistant *Enterobacterales* and P. aeruginosa producing variant KPC and PDC enzymes (9).

The epidemiology of carbapenem resistance mechanisms in *Enterobacterales* and P. aeruginosa is important to new agent development. Cefepime-taniborbactam at a concentration of 16 μg/mL inhibited 94.5% (563/596 with carbapenemase) and 95.1% (39/41 without carbapenemase) of CRE and 71.1% (177/249 with carbapenemase) and 94.8% (714/753 without carbapenemase) of CRPA in the present study. The somewhat reduced activity of cefepime-taniborbactam against carbapenemase-positive CRPA may be partly explained by IMP expression in some isolates and by the presence of NDM, which in the presence of elevated efflux or PBP3 alterations, likely increases cefepime MICs too high to be restored to ≤16 μg/mL by taniborbactam. While carbapenemase production is a less frequent mechanism of resistance in CRPA in many regions, cefepime-taniborbactam inhibited 87.4% (139/159) of CRPA isolates producing VIM, the most common carbapenemase in CRPA.

The current study has at least three limitations. First, the identification of resistance mechanisms was based solely on whole-genome sequencing (WGS) data from isolates with elevated cefepime-taniborbactam MICs. Complementation studies and genotyping of strains with cefepime-taniborbactam MICs of ≤8 μg/mL would be required to establish genotypic-phenotypic relationships. Second, we did not directly assess efflux pump or β-lactamase expression, which is known to confer increased MICs to most classes of agents in *Enterobacterales* and P. aeruginosa and may affect cefepime-taniborbactam activity (7). Third, the study only included β-lactam/β-lactamase inhibitor combinations that had been approved for clinical use in 2018 when the surveillance study was initiated. For this reason, neither aztreonam-avibactam nor imipenem-relebactam was included. However, there is substantial overlap between the activities of meropenem-vaborbactam (included in this study) and imipenem-relebactam in that both β-lactam/β-lactamase inhibitor combinations are carbapenem based and the respective β-lactamase inhibitors have largely overlapping spectra of enzyme inhibition (e.g., KPC and AmpC, but not OXA-48 or metallo-β-lactamases).

In conclusion, taniborbactam restored cefepime activity against CRE and CRPA, including in isolates carrying serine- and metallo-β-lactamases and in isolates without carbapenemases. These findings support continued development of cefepime-taniborbactam as a potential new therapeutic agent for patients with MDR or DTR infections.

## MATERIALS AND METHODS

### Bacterial isolates.

Clinical isolates of *Enterobacterales* (*n *= 13,731) and P. aeruginosa (*n *= 4,619) were cultured from patients with community- and hospital-associated infections in 264 sites in 56 countries across 7 geographic regions in the years 2018 (*n *= 5,912), 2019 (*n *= 6,933), and 2020 (*n *= 5,505). The isolates were shipped to IHMA (Schaumburg, IL, USA), where their identities were confirmed using matrix-assisted laser desorption ionization–time of flight (MALDI-TOF) mass spectrometry (Bruker Daltonics, Billerica, MA, USA). Isolates were limited to one per patient. The composition of *Enterobacterales* isolates by species is shown in Table S6. The distribution of isolates by geographic region is shown in Table S7.

### Antimicrobial susceptibility testing.

MIC values were determined using the CLSI broth microdilution reference method with concurrent quality control (30, 31). Taniborbactam was provided by Venatorx Pharmaceuticals, Inc. (Malvern, PA, USA) and tested at a fixed concentration of 4 μg/mL with cefepime (31). Other agents were purchased from commercial sources. Broth microdilution panels were prepared at IHMA using cation-adjusted Mueller Hinton broth (CAMHB; Becton, Dickinson) and stored at −70°C until the day of testing.

MICs were interpreted using 2021 CLSI breakpoints (31) with two exceptions. Meropenem-vaborbactam MICs for P. aeruginosa were interpreted using EUCAST breakpoints (≤8 μg/mL, susceptible; >8 μg/mL, resistant) (32). Cefepime-taniborbactam MICs against both *Enterobacterales* and P. aeruginosa were interpreted using provisional breakpoints of ≤16 μg/mL (susceptible) and >16 μg/mL (resistant) that were based upon published *in vivo* efficacy data from neutropenic murine infection models (thigh, complicated urinary tract) (33, 34) and data from a safety and pharmacokinetics studies in human volunteers (35, 36). These provisional breakpoints are also supported by probability of taniborbactam target attainment analyses based on simulation of human plasma exposures (J. Dowell, unpublished data).

### Molecular testing.

WGS was performed on an Illumina HiSeq platform using 2 × 150-bp paired-end reads with a target coverage depth of 100× for all isolates of *Enterobacterales* (*n *= 74) and P. aeruginosa (*n *= 267) with cefepime-taniborbactam MICs of ≥16 μg/mL. All sequence analyses were carried out using the CLC Genomics Workbench version 20 (Qiagen). Antimicrobial resistance genes were identified in *de novo* assemblies of each genome using the “find resistance” module in the Center for Genomic Epidemiology (CGE) database for resistance genes (37). The major porin genes *ompF* and *ompC* (*ompK35* and *ompK36* in K. pneumoniae) in *Enterobacterales* and *oprD* in P. aeruginosa were identified using tBLASTn and screened for alterations. Disrupted genes were defined as those (i) carrying any mutation that caused a stop codon to be read in-frame upstream of the stop codon in the reference sequence and/or (ii) on different contigs and interspaced by an insertion sequence (as identified by a BLAST search in the GenBank nonredundant/nucleotide [nr/nt] database). Insertions and/or deletions that did not alter the porin gene reading frame were not considered disruptions, with the exception of variation in the L3 internal loop that constitutes the channel eyelet in OmpK36, including mutations that coded for the insertion of an Asp, Gly-Asp, Asp-Ser, Asp-Thr, or Ser-Asp residue(s) in this region (38, 39). The sole amino acid substitution considered a disruption was the Gly137Asp variation in the L3 loop of OmpC associated with reduced susceptibility to cephalothin, cefoxitin, moxalactam, and ertapenem (17). For *ftsI* (encoding PBP3), efflux pump regulatory gene, and other gene-specific analyses, appropriate NCBI protein reference sequences were searched using BLAST on a species-specific basis.

Isolates with cefepime-taniborbactam MICs of <16 μg/mL, but resistant to meropenem (*Enterobacterales* MIC, ≥4 μg/mL; P. aeruginosa MIC, ≥8 μg/mL) (*n *= 573 *Enterobacterales*; *n *= 732 P. aeruginosa) were screened for acquired β-lactamase genes by PCR, followed by Sanger sequencing as previously described (40). Additionally, 614 randomly selected *Enterobacterales* with cefepime and/or ceftazidime MIC values of ≥2 μg/mL and 92 randomly selected P. aeruginosa with ceftazidime and/or cefepime MIC values of ≥16 μg/mL were also screened by PCR, followed by Sanger sequencing, for the following β-lactamase genes as previously described: ESBLs (CTX-M, GES, PER, SHV, TEM, VEB), acquired AmpC β-lactamases (ACC, ACT, CMY, DHA, FOX, MIR, MOX), serine carbapenemases (GES, KPC, OXA-48-like [*Enterobacterales*], OXA-24-like [P. aeruginosa]), and MBLs (GIM, IMP, NDM, SPM, VIM) (40, 41). Also, to better understand the association between PBP3 mutations and cefepime-taniborbactam MICs, randomly selected isolates of E. coli (*n *= 80) and P. aeruginosa (*n *= 150) with MICs of ≤8 μg/mL were chosen from study isolates for *ftsI* gene PCR amplification/Sanger sequencing or WGS (Additional Study S1).
