# Supplementary material for: In Vitro Activity of Cefepime-Taniborbactam and Comparators against Clinical Isolates of Gram-Negative Bacilli from 2018 to 2020: Results from the Global Evaluation of Antimicrobial Resistance via Surveillance (GEARS) Program
Source: Antimicrob Agents Chemother. 2022 Dec 21;67(1):e01281-22. doi: 10.1128/aac.01281-22 (PMC9872668; doi:10.1128/aac.01281-22)
Supplement: Supplemental file 1 — Supplemental material. Download aac.01281-22-s0001.pdf, PDF file, 0.2 MB [file aac.01281-22-s0001.pdf]

SUPPLEMENTARY DATA

TABLE S1 *In vitro* activity of cefepime-taniborbactam and comparator agents against 13,731 isolates of Enterobacterales

	MIC, µg/ml			MIC interpretation		
Antimicrobial agent	MIC_{50}	MIC_{90}	MIC range	% susceptible	% intermediate	% resistant
Cefepime-taniborbactam[a]	0.06	0.25	≤0.008 - >16	99.7	NA[b]	0.3
Cefepime	≤0.25	>16	≤0.25 - >16	77.3	NA	17.4
Ceftazidime-avibactam	≤0.12	0.5	≤0.12 - >16	97.8	NA	2.2
Ceftazidime	0.5	>16	≤0.03 - >16	74.2	2.3	23.4
Ceftolozane-tazobactam	0.5	8	≤0.25 - >8	87.1	2.4	10.5
Gentamicin	0.5	>16	≤0.12 - >16	83.8	0.8	15.4
Levofloxacin	0.12	>8	≤0.004 - >8	69.8	4.2	26.0
Meropenem-vaborbactam	≤0.06	0.12	≤0.06 - >16	97.4	0.3	2.2
Meropenem	0.03	0.12	≤0.004 - >64	94.8	0.6	4.6
Piperacillin-tazobactam	≤4	128	≤4 - >128	84.2	4.9	10.9

[a] For comparative purposes only, % susceptible and % resistant for cefepime-taniborbactam correspond to % of isolates inhibited at ≤16 µg/ml and ≥32 µg/ml, respectively.
[b] NA, not applicable.

TABLE S2 *In vitro* activity of cefepime-taniborbactam and cefepime against individual species of Enterobacterales

Species[a] (no. of isolates)	Antimicrobial agent	MIC, µg/ml			MIC interpretation		
		MIC_{50}	MIC_{90}	MIC range	% susceptible	% intermediate	% resistant
Citrobacter freundii complex (1,126)	Cefepime-taniborbactam[b]	0.03	0.12	≤0.008 - >16	99.9	NA[c]	0.1
	Cefepime	≤0.25	2	≤0.25 - >16	93.3	1.9	4.9
Enterobacter cloacae complex (797)	Cefepime-taniborbactam	0.06	0.5	≤0.008 - >16	99.9	NA	0.1
	Cefepime	≤0.25	16	≤0.25 - >16	78.3	10.3	11.4
Escherichia coli (4,041)	Cefepime-taniborbactam	0.03	0.12	≤0.008 - >16	99.7	NA	0.3
	Cefepime	≤0.25	>16	≤0.25 - >16	74.3	7.4	18.3
Klebsiella aerogenes (390)	Cefepime-taniborbactam	0.06	0.12	0.016 - 4	100	NA	0
	Cefepime	≤0.25	0.5	≤0.25 - >16	96.2	2.6	1.3
Klebsiella oxytoca (759)	Cefepime-taniborbactam	0.03	0.06	≤0.008 - 4	100	NA	0
	Cefepime	≤0.25	1	≤0.25 - >16	94.5	3.6	2.0
Klebsiella pneumoniae (4,024)	Cefepime-taniborbactam	0.06	1	≤0.008 - >16	99.5	NA	0.5
	Cefepime	≤0.25	>16	≤0.25 - >16	62.0	5.1	33.0
Morganella morganii (389)	Cefepime-taniborbactam	0.03	0.12	≤0.008 - 1	100	NA	0
	Cefepime	≤0.25	≤0.25	≤0.25 - >16	96.7	1.5	1.8
Proteus mirabilis (758)	Cefepime-taniborbactam	0.06	0.25	≤0.008 - 8	100	NA	0
	Cefepime	≤0.25	16	≤0.25 - >16	84.8	4.8	10.4
Proteus vulgaris (358)	Cefepime-taniborbactam	0.06	0.06	0.016 - 8	100	NA	0
	Cefepime	≤0.25	≤0.25	≤0.25 - >16	98.6	0.8	0.6
Providencia rettgeri (164)	Cefepime-taniborbactam	0.016	0.12	≤0.008 - >16	98.2	NA	1.8
	Cefepime	≤0.25	0.5	≤0.25 - >16	92.7	3.1	4.3
Providencia stuartii (213)	Cefepime-taniborbactam	0.06	0.12	0.016 - 2	100	NA	0
	Cefepime	≤0.25	8	≤0.25 - >16	82.6	8.0	9.4
Serratia liquefaciens (58)	Cefepime-taniborbactam	0.06	0.12	≤0.008 – 0.5	100	NA	0
	Cefepime	≤0.25	≤0.25	≤0.25 – 0.5	100	0	0

Serratia marcescens (530)	Cefepime-taniborbactam	0.06	0.25	0.016 - >16	99.6	NA	0.4
	Cefepime	≤0.25	2	≤0.25 - >16	90.4	1.3	8.3

[a] Species/groups listed were limited to those with 50 or more isolates tested.
[b] For comparative purposes only, % susceptible and % resistant for cefepime-taniborbactam correspond to % of isolates inhibited at ≤16 µg/ml and ≥32 µg/ml, respectively.
[c] NA, not applicable.

TABLE S3 Cefepime-taniborbactam MIC distribution for 627 isolates of carbapenemase-positive Enterobacterales[a] stratified by carbapenemase type and susceptibility to cefepime

		Cefepime-taniborbactam MIC, µg/ml (% of isolates at MIC)											
		0.03	0.06	0.12	0.25	0.5	1	2	4	8	16	≥32	Total
Carbapenemase	KPC	9 (75.0)	27 (100)	33 (68.8)	34 (51.5)	29 (32.2)	40 (32.3)	31 (29.5)	16 (25.8)	9 (27.3)	0 (0)	0 (0)	228
	NDM	0 (0)	0 (0)	4 (8.3)	11 (16.7)	45 (50.0)	43 (34.7)	29 (27.6)	7 (11.3)	15 (45.5)	25 (89.3)	28 (93.3)	207
	OXA-48-like	2 (16.7)	0 (0)	10 (20.8)	18 (27.3)	12 (13.3)	33 (26.6)	44 (41.9)	38 (61.3)	8 (24.2)	1 (3.6)	2 (6.6)	168
	VIM	1 (8.3)	0 (0)	1 (2.1)	3 (4.5)	4 (4.4)	8 (6.5)	1 (1.0)	1 (1.6)	1 (4.2)	2 (7.1)	0 (0)	22
	Total	12 (100)	27 (100)	48 (100)	66 (100)	90 (100)	124 (100)	105 (100)	62 (100)	33 (100)	28 (100)	30 (100)	625[b]
	Cum. % inhibited[c]	1.9	6.2	13.9	24.5	38.9	58.7	75.5	85.4	90.7	95.2	100	
Cefepime	MIC_{50}	8	16	≥32	≥32	≥32	≥32	≥32	≥32	≥32	≥32	≥32	
	MIC_{90}	≥32	≥32	≥32	≥32	≥32	≥32	≥32	≥32	≥32	≥32	≥32	
	% susceptible	33.3	7.4	6.3	9.1	3.3	0.8	0	0	0	0	0	
	% SDD[d]	41.7	22.2	14.6	4.5	2.2	0.8	1.0	0	0	0	0	
	% resistant	25.0	70.4	79.2	86.4	94.4	98.4	99.0	100	100	100	100	

[a] IMP-positive isolates (*n*=6) were excluded from the dataset as IMP is outside the spectrum of taniborbactam inhibition.
[b] Two isolates tested with cefepime-taniborbactam MICs of 0.016 µg/ml (2 KPC-positive isolates); these isolates were excluded from the table.
[c] Cumulative % of all carbapenemase-positive isolates inhibited at cefepime-taniborbactam MIC.
[d] SDD, susceptible-dose dependent.

TABLE S4. *In vitro* activity of cefepime-taniborbactam and comparator agents against 4,619 isolates of *P. aeruginosa*

	MIC, µg/ml			MIC interpretation		
Antimicrobial agent	MIC_{50}	MIC_{90}	MIC range	% susceptible	% intermediate	% resistant
Cefepime-taniborbactam[a]	2	8	≤0.06 - >32	97.4	NA[b]	2.6
Cefepime	4	32	≤0.25 - >32	79.4	9.0	11.7
Ceftazidime-avibactam	2	8	≤0.25 - >16	90.5	NA	9.6
Ceftazidime	4	>32	≤0.25 - >32	76.9	4.7	18.4
Ceftolozane-tazobactam	1	8	≤0.12 - >16	88.8	2.6	8.7
Ciprofloxacin	0.12	>4	≤0.06 - >4	74.7	4.1	21.2
Gentamicin	2	>16	≤0.25 - >16	83.7	4.1	12.2
Imipenem	2	>8	≤0.5 - >8	53.8	15.5	30.6
Meropenem-vaborbactam[c]	0.5	16	≤0.06 - >16	86.6	NA	13.4
Meropenem	0.5	>8	≤0.06 - >8	73.5	5.9	20.5
Piperacillin-tazobactam	8	>128	≤0.5 - >128	70.8	14.2	15.0

[a] For comparative purposes only, % susceptible and % resistant for cefepime-taniborbactam correspond to % of isolates inhibited at ≤16 µg/ml and ≥32 µg/ml, respectively.
[b] NA, not applicable.
[c] Meropenem-vaborbactam MICs for *P. aeruginosa* were interpreted using EUCAST breakpoints (≤8 µg/ml, susceptible; >8 µg/ml, resistant) (EUCAST v11, 2021) because CLSI has not published breakpoints for this agent-organism combination.

TABLE S5 Cefepime-taniborbactam MIC distribution for 216 isolates of carbapenemase-positive *P. aeruginosa*[a] stratified by carbapenemase type and susceptibility to cefepime

		Cefepime-taniborbactam MIC, µg/ml (% of isolates at MIC)					
		≤4	8	16	32	≥64	Total
Carbapenemase	VIM	38 (84.4)	82 (77.4)	19 (73.1)	5 (55.6)	15 (50.0)	159
	GES	7 (15.5)	21 (19.8)	5 (19.2)	0 (0)	0 (0)	33
	NDM	0 (0)	0 (0)	0 (0)	3 (33.3)	14 (46.7)	17
	KPC	0 (0)	3 (2.8)	2 (7.7)	1 (11.1)	1 (3.3)	7
	Total	45 (100)	106 (100)	26 (100)	9 (100)	30 (100)	216
	Cum. % inhibited[b]	20.8	69.9	81.9	86.1	100	
Cefepime	MIC_{50}	16	≥32	≥32	≥32	≥32	
	MIC_{90}	≥32	≥32	≥32	≥32	≥32	
	% susceptible	20.0	16.0	0	0	0	
	% intermediate	40.0	30.2	19.2	0	0	
	% resistant	40.0	53.8	80.8	100	100	

[a] IMP-positive isolates (*n*=33) were excluded from the dataset as IMP is outside the spectrum of taniborbactam inhibition.

[b] Cumulative % of all carbapenemase-positive isolates inhibited at cefepime-taniborbactam MIC.

TABLE S6 Total isolate counts by group/species of Enterobacterales

Group/Species	Total isolates (% of total)
Citrobacter freundii complex[a]	1,126 (8.2)
Enterobacter cloacae complex[b]	797 (5.8)
Escherichia coli	4,041 (29.4)
Klebsiella aerogenes	390 (2.8)
Klebsiella oxytoca	759 (5.5)
Klebsiella pneumoniae	4,024 (29.3)
Klebsiella variicola	4 (<0.1)
Morganella morganii	389 (2.8)
Proteus mirabilis	758 (5.5)
Proteus vulgaris	358 (2.6)
Providencia alcalifaciens	3 (<0.1)
Providencia rettgeri	164 (1.2)
Providencia sp.	2 (<0.1)
Providencia stuartii	213 (1.6)
Serratia fonticola	1 (<0.1)
Serratia liquefaciens	58 (0.4)
Serratia marcescens	530 (3.9)
Serratia odorifera	2 (<0.1)
Serratia rubidaea	5 (<0.1)
Serratia sp.	83 (0.6)
Serratia ureilytica	24 (0.2)
Total	13,731 (100)

[a] *Citrobacter freundii* complex isolates consisted of: 681 *Citrobacter freundii*, 374 *Citrobacter koseri*, 53 *Citrobacter braakii*, 7 *Citrobacter amalonaticus*, 4 *Citrobacter farmeri*, 4 *Citrobacter* sp., 2 *Citrobacter sedlakii*, and 1 *Citrobacter youngae*.

[b] *Enterobacter cloacae* complex isolates consisted of: 586 *Enterobacter cloacae*, 54 *Enterobacter asburiae*, 27 *Enterobacter bugandensis*, 24 *Enterobacter xiangfangensis*, 23 *Enterobacter* sp.,17 *Enterobacter kobei*, 8 *Enterobacter ludwigii*, and 58 *Enterobacter cloacae* complex, unable to further speciate.

TABLE S7 Total isolate counts of Enterobacterales and *P. aeruginosa* by global region of origin

Region of origin	Enterobacterales (% of total isolates)	*P. aeruginosa* (% of total isolates)
Africa	428 (3.1)	183 (4.0)
Asia	1,289 (9.4)	363 (7.9)
Europe	5,809 (42.3)	2,035 (44.1)
Latin America	1,534 (11.2)	573 (12.4)
Middle East	464 (3.4)	271 (5.9)
North America	3,873 (28.2)	1,044 (22.6)
South Pacific	334 (2.4)	150 (3.2)
Total	13,731 (100)	4,619 (100)

ADDITIONAL STUDY S1 *ftsI* (PBP3) analysis

To better understand the association between PBP3 sequence mutations and cefepime-taniborbactam MICs, additional isolates of *E. coli* (*n*=92) and *P. aeruginosa* (*n*=299) with MICs ≤16 μg/ml (*E. coli*, MIC range, 0.03–16 μg/ml; *P. aeruginosa*, MIC range, 0.25–16 μg/ml) were chosen from study isolates for *ftsI* gene PCR amplification/Sanger sequencing or WGS. For *E. coli*, 27 of the 92 isolates (29.3%) with cefepime-taniborbactam MICs ≤16 μg/ml exhibited either a YRIN or YRIK insertion at position 333 in PBP3. In comparison, 73.7% (28/38) of all *E. coli* isolates identified in the main study with one of these four amino acid insertions in PBP3 had a cefepime-taniborbactam MIC of ≤16 μg/ml. In the main study, 10 isolates tested with cefepime-taniborbactam MICs >16 μg/ml, 11 isolates tested with cefepime-taniborbactam MICs of 16 μg/ml, and 17 isolates tested with cefepime-taniborbactam MICs ≤8 μg/ml. Therefore, it appears that such an insertion alone is insufficient to consistently elevate cefepime-taniborbactam MICs above 8 μg/ml. However, this mutation in combination with an acquired β-lactamase, particularly NDM, may be sufficient to significantly increase the cefepime-taniborbactam MIC, as 17 of the 21 isolates with this PBP3 insertion, that tested with a cefepime-taniborbactam MIC of ≥16 μg/ml, also harbored NDM. For *P. aeruginosa*, PBP3 amino acid variants previously shown to have an effect on β-lactam activity (G63D, G216S, A244T, R504C, I524T, P527S, G531D, F533L) were not observed in any of the isolates tested with cefepime-taniborbactam MICs ≤8 μg/ml, whereas 20 of 50 isolates (40.0%) with one of these specific variants were inhibited at 16 μg/ml cefepime-taniborbactam (13.4% [20/149] of all isolates testing with a cefepime-taniborbactam MIC of 16 μg/ml) and the remaining 30 isolates at >16 μg/ml (25.4% [30/118] of all isolates tested with a cefepime-taniborbactam MIC of >16 μg/ml).
